# Supplementary material for: Predicting visual function by interpreting a neuronal wiring diagram
Source: Nature. 2024 Oct 2;634(8032):113–23. doi: 10.1038/s41586-024-07953-5 (PMC11446822; doi:10.1038/s41586-024-07953-5)
Supplement: Supplementary file 6 — CRF and ERF predictions for individual TmY4 and TmY9 cells. Analogous to Supplementary Data 3, but for TmY target types. Shown are the top four monosynaptic pathways, the strongest pathway passing through each of the top ten intermediary types (ranking from Extended Data Fig. 7), and the trisynaptic pathway Tm1–TmY–Dm3–TmY (see the section entitled Prediction of spatial normalization). [file 41586_2024_7953_MOESM6_ESM.zip › DataS4/TmY9q/720575940625124583.pdf]

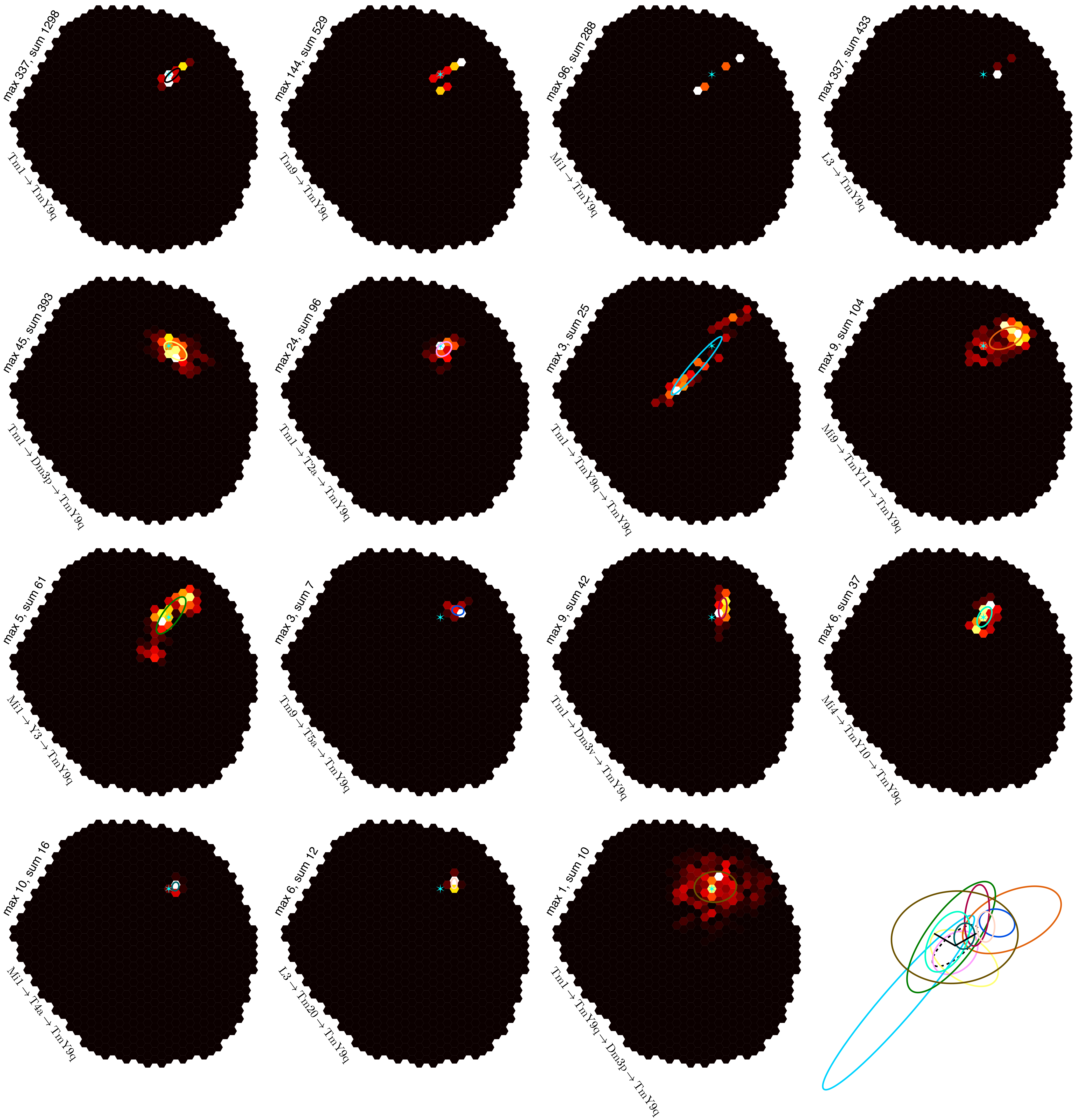
max 337, sum 1298
Tm1 → TmY9q
max 144, sum 529
Tm9 → TmY9q
max 96, sum 288
Mi1 → TmY9q
max 337, sum 433
L3 → TmY9q
max 45, sum 393
Tm1 → Dm3p → TmY9q
max 24, sum 96
Tm1 → T2a → TmY9q
max 3, sum 25
Tm1 → TmY9q → TmY9q
max 9, sum 104
Mi9 → TmY11 → TmY9q
max 5, sum 61
Mi1 → Y3 → TmY9q
max 3, sum 7
Tm9 → T5a → TmY9q
max 9, sum 42
Tm1 → Dm3v → TmY9q
max 6, sum 37
Mi4 → TmY10 → TmY9q
max 10, sum 16
Mi1 → T4a → TmY9q
max 6, sum 12
L3 → Tm20 → TmY9q
max 1, sum 10
Tm1 → TmY9q → Dm3p → TmY9q
